# Assessment of the Abnormalities in Chest Computed Tomography and Pulmonary Function Test in Convalescents Six Months After COVID-19

**DOI:** 10.3390/medicina61050823

**Published:** 2025-04-29

**Authors:** Katarzyna Guziejko, Anna Moniuszko-Malinowska, Robert Flisiak, Piotr Czupryna, Sebastian Sołomacha, Paweł Sowa, Marlena Dubatówka, Magda Łapińska, Łukasz Kiszkiel, Łukasz Szczerbiński, Piotr Paweł Laskowski, Maciej Alimowski, Gabriela Trojan, Karol Adam Kamiński

**Affiliations:** 1 2nd Department of Lung Diseases, Lung Cancer and Internal Medicine, Medical University of Bialystok, 15-089 Bialystok, Poland; 2 Department of Infectious Diseases and Neuroinfections, Medical University of Bialystok, 15-089 Bialystok, Poland; avalon-5@wp.pl; 3 Department of Infectious Diseases and Hepatology, Medical University of Bialystok, 15-089 Bialystok, Poland; robert.flisiak1@gmail.com; 4 Department of Population Medicine and Lifestyle Diseases Prevention, Medical University of Bialystok, 15-089 Bialystok, Poland; sebastian.solomacha@umb.edu.pl (S.S.); mailtosowa@gmail.com (P.S.); marlena.dubatowka@umb.edu.pl (M.D.); magda.lapinska@umb.edu.pl (M.Ł.); 5 Society and Cognition Unit, University of Bialystok, 15-328 Bialystok, Poland; lukaszkiszkiel@gmail.com (Ł.K.); pio.laskowski@gmail.com (P.P.L.); 6 Clinical Research Centre, Medical University of Bialystok, 15-089 Bialystok, Poland; sz.lukasz91@gmail.com; 7 Department of Endocrinology, Diabetology and Internal Medicine, Medical University of Bialystok, 15-089 Bialystok, Poland; 8 Doctoral School of Social Sciences, University of Bialystok, 15-328 Bialystok, Poland; m.alimowski@uwb.edu.pl

**Keywords:** COVID-19, chest CT, body plethysmography, DLCO, dynamic spirometry

## Abstract

*Background:* Despite the multiple waves of the COVID-19 pandemic, follow-up strategies for recovered patients remain inconclusive. This study aimed to evaluate chest computed tomography (CT) and pulmonary function test (PFT) abnormalities in convalescents six months after COVID-19 and to compare these findings with those from a representative population cohort. The goal was to support more individualized pulmonary management of post-COVID-19 sequelae. *Methods*: This study population consisted of 2 groups: I—232 post-COVID-19 patients and II—543 patients from a population cohort. Chest CT was performed during the acute phase of COVID-19 and six months after. The PFTs were conducted six months after COVID-19. *Results*: There were no significant differences in FEV1, FVC, TLC, and DLCO in the two study groups. A singular GGO in 24 patients (20%), a crazy paving pattern in 1 patient (0.8%), thickening of interlobular septa in 4 patients (3.5%), consolidations in 4 patients (3.5%), traction bronchiectasis in 6 patients (5%), fibrosis in 6 patients (5%), and singular nodular densities in 68 patients (58%) were observed in chest CT 6 months after COVID-19. Most radiological abnormalities were clinically insignificant and did not require further diagnostic evaluation. No significant differences in chest CT and PFT six months after infection were observed between patients differing in the severity of inflammation during the acute disease or SARS-CoV-2 variant. *Conclusions*: The majority of chest CT abnormalities resolved within six months of recovery, regardless of SARS-CoV-2 variant or initial disease severity. Pulmonary function tests should be prioritized in post-COVID-19 follow-up, as PFT results in convalescents were comparable to those observed in the general population.

## 1. Introduction

Numerous studies and meta-analyses worldwide have examined the long-term pulmonary sequelae in patients following COVID-19 pneumonia [1,2,3,4,5,6,7,8,9,10,11,12,13]. However, their findings remain inconclusive. Persistent clinical symptoms, radiological abnormalities, and functional impairments of the respiratory system vary significantly depending on the study design, duration of follow-up, and characteristics of the observed cohorts [6,7,8,9,10,13]. Pulmonary function tests—including dynamic spirometry, body plethysmography, and diffusing capacity for carbon monoxide (DLCO)—along with chest computed tomography (CT), form the cornerstone of comprehensive assessment of respiratory complications following COVID-19 [1,3,5,6,9,10,11,12]. Increasing awareness of the clinical value of follow-up in COVID-19 survivors allows for more personalized planning of post-COVID-19 management strategies [4,8,9,10,11,12]. Our previous study demonstrated that not all persistent symptoms observed six months after SARS-CoV-2 infection are associated with impaired pulmonary function [13]. However, patients presenting with chronic cough may exhibit abnormalities in pulmonary function tests (PFTs) [13]. In the present study, we aimed to expand our assessment by including chest imaging results from a larger cohort of patients hospitalized due to COVID-19 and comparing their pulmonary function tests with those of the general population.

Therefore, the current study aimed to assess the results of chest CT and functional assessment of the respiratory system based on spirometry, body plethysmography, and DLCO 6 months after COVID-19 during the four waves of the pandemic in comparison to the general population.

Detailed aims were as follows:

- To assess the results of chest CT in patients six months after infection and compare them with baseline examinations performed during the acute phase during four waves of the pandemic.
- To assess the results of respiratory function tests in patients six months after infection and compare them with the general population during four waves of the pandemic.
- To compare the results of baseline chest CT and respiratory function tests performed six months after COVID-19 in relation to the severity of inflammation during the acute disease stratified by laboratory tests, including interleukin 6, c-reactive protein (CRP), and d-dimer concentration.
- To assess the correlation between abnormalities of baseline chest CT and pulmonary function tests performed six months after COVID-19 with selected laboratory parameters during the acute phase of the disease.

## 2. Materials and Methods

The study population comprised 775 participants (365 men and 410 women), with a mean age of 52 years (range: 20–80). Participants were divided into two groups. Group I (post-COVID-19) included 232 patients with a confirmed SARS-CoV-2 infection (positive PCR test), all of whom required hospitalization during the acute phase and consented to follow-up examinations six months after discharge. Patients in Group I were hospitalized at the Department of Infectious Diseases, Medical University of Bialystok Clinical Hospital, or in a temporary hospital established for COVID-19 care. Participants in Group I were further divided into six subgroups based on inflammatory markers measured during the acute phase of infection:

- Ia: IL-6 > 100 pg/mL (*n* = 36).
- Ib: IL-6 < 100 pg/mL (*n* = 111).
- Ic: PLT > 150 × 10^3^/µL (*n* = 129).
- Id: PLT < 150 × 10^3^/µL (*n* = 47).
- Ie: d-dimer < 1000 ng/mL (*n* = 108).
- If: d-dimer > 1000 ng/mL (*n* = 61).

Demographic characteristics, selected laboratory findings during hospitalization, and the treatment applied are presented in Table 1.

Baseline chest computed tomography (CT) was performed during the acute phase of COVID-19. Follow-up imaging was conducted approximately six months after recovery in 118 participants. All CT scans were evaluated by an experienced radiologist. The assessment focused on features typically associated with COVID-19, including ground-glass opacities (GGOs), crazy paving patterns, consolidations, reticular changes, thickening of interlobular septa, and traction bronchiectasis, as well as the distribution and predilection of findings.

The extent of lung parenchymal involvement was quantified either as a percentage of affected tissue or by using a CT severity score (ranging from 0 to 25), calculated by summing individual lobe scores (0–5 per lobe) [14].

In addition, baseline CT findings were analyzed in relation to inflammatory parameters, including interleukin-6 (IL-6), platelet count (PLT), and d-dimer levels, in this group of patients.

The clinical course of COVID-19 in Group I ranged from mild to moderately severe. Notably, none of the participants required intensive care unit (ICU) admission or mechanical ventilation during the acute phase of the disease.

Group II included 543 individuals (250 men and 293 women; median age 50 years, range: 20–80) from the population-based Bialystok PLUS cohort study. This cohort was randomly selected from the local community, making it representative in terms of demographic and health-related characteristics. This study evaluates the health status of the population through clinical examinations and standardized questionnaires, offering insights into medical, sociological, and psychological aspects of health [15,16]. Participants in Group II were enrolled between December 2020 and September 2022 and were selected to match Group I in terms of age, sex, and body mass index (BMI). Among them, 262 individuals (48%) tested positive for anti-N IgG antibodies. According to self-reports, these infections were asymptomatic or mild, did not require medical attention, and were not clinically diagnosed.

Although some participants had serological evidence of prior SARS-CoV-2 infection, this cohort was intended to reflect the general population background at the time of this study—including undiagnosed or subclinical cases. As such, it served as a practical and relevant comparison group for assessing long-term pulmonary outcomes following COVID-19 hospitalization.

Both study groups were considered representative of the local population.

All participants underwent pulmonary function testing, including dynamic spirometry, body plethysmography, and diffusing capacity for carbon monoxide (DLCO), using the BodyBox 5500 system (Medisoft, Belgium). All procedures were conducted in accordance with current international guidelines [17]. The following parameters were included in the analysis: FEV_1_ (forced expiratory volume in 1 s), FVC (forced vital capacity), FEV_1_/FVC ratio, TLC (total lung capacity), and DLCO (corrected for hemoglobin). Pulmonary impairment was defined as a value below 70% of the predicted value for any of the assessed parameters (Table 2).

Pulmonary function tests and follow-up chest CT scans were performed six months after initial hospitalization. The four pandemic waves were defined based on the dominant circulating SARS-CoV-2 variants in the region:

- Wave 1 (29 February 2020–31 December 2020): Wild-type variants (*n* = 114).
- Wave 2 (1 January 2021–30 April 2021): Wild-type and Alpha variants (*n* = 121).
- Wave 3 (1 May 2021–31 December 2021): Delta variant (*n* = 222).
- Wave 4 (1 January 2022–31 March 2022): Omicron variant (*n* = 318).

Participants also completed a health history and COVID-19 vaccination questionnaire (Table 3).

This study received ethical approval from the Ethics Committee of the Medical University of Bialystok, Poland (approval number: APK.002.346.2020). All procedures were conducted in accordance with the Declaration of Helsinki. Participation in this study was voluntary, and written informed consent was obtained from all participants prior to the initiation of any study-related procedures. Detailed information regarding all procedures was provided to each participant before enrollment.

Exclusion criteria included contraindications to respiratory function tests (e.g., hemoptysis of unknown origin, unstable cardiovascular status, recent myocardial infarction or pulmonary embolism, recent eye surgery, etc.) and chest computed tomography (e.g., claustrophobia, allergy to the intravenous contrast media, etc.).

The statistical analysis was performed using the Statistica 13.0 program. Data were presented as medians, minimums, and maximums, as appropriate. The normal distribution was evaluated by the Shapiro–Wilk test. In statistical analysis, the Kruskal-Wallis test and the chi-squared test were used. A probability level of *p* < 0.05 was considered statistically significant.

## 3. Results

### 3.1. Comparison of Results of Chest CT Between Symptomatic Patients During the Acute Phase and Six Months After Infection 

**Figure 1:**
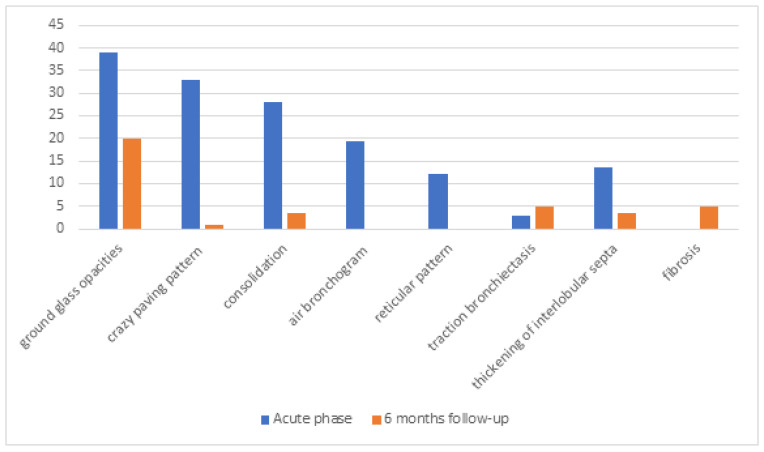
The results of chest computed tomography in symptomatic patients during the acute phase and 6 months of follow-up after infection. Values are expressed as percentages.

At baseline imaging, GGO was observed in 46 patients (39%), crazy paving pattern in 39 patients (33%), consolidation in 33 patients (28%), air bronchogram in 23 patients (19.5%), reticular pattern in 14 patients (12%), traction bronchiectasis in 4 patients (3%), thickening of interlobular septa in 16 patients (13.5%), bilateral distribution in 37 patients (31%), peripheral distribution in 6 patients (5%), and predilection to lower lobes on the right in 31 patients (26%) and left in 29 patients (24.5%) sides (shown in Figure 1).

The median lung parenchyma involvement was rated at 27.5% or scored 8/25 points. Follow-up imaging revealed the presence of a single ground-glass opacity (GGO) in 24 patients (20%), a crazy paving pattern in 1 patient (0.8%), thickening of interlobular septa in 4 patients (3.5%), consolidations in 4 patients (3.5%), traction bronchiectasis in 6 patients (5%), fibrosis in 6 patients (5%), singular small nodular densities in 68 patients (58%), localized bilaterally in 50 patients (42%), peripherally in 43 patients (36.5%), and at posterior segments in 44 patients (37%) (shown in Figure 1). Except for one patient with pulmonary fibrosis, most radiological findings were clinically insignificant and required no additional diagnostic procedures. In follow-up chest CT scans, only 2 patients (1.7%) had significant involvement of the lung parenchyma in the radiologist’s opinion. In the first patient, lung parenchyma involvement was assessed as 1% of total lungs, and in the second patient, it was assessed with a score of 17/25 points. In the first case, GGO, air bronchogram, and reticular densities persist bilaterally in the lower lobes. In the second case, diffused GGO and reticular densities persist bilaterally in all lobes, with no apparent predilection for peripheral or central areas. In the clinical assessment, the second patient has diminished exercise tolerance, periodically uses passive oxygen therapy (FiO_2_–0.28), and is under pulmonological follow-up (Table 4). Revealed abnormalities were statistically insignificant in relation to baseline assessment. Additionally, no lung nodules were observed in the study group.

### 3.2. Comparison of Results of Pulmonary Function Tests Between Patients Six Months After SARS-CoV-2 Infection and the General Population

There were no significant differences in FEV1, FVC, TLC, and DLCO between the two study groups, regardless of the wave of the pandemic (Table 2).

### 3.3. Impact of Laboratory Test Results on Chest CT Findings During the Acute Phase of COVID-19

No significant differences in chest CT were observed regarding the severity of inflammation during the acute disease stratified by laboratory tests, including IL-6, CRP, and d-dimer concentration (Table 5).

### 3.4. Comparison of Results of Pulmonary Function Tests Between Post-COVID-19 Patients and the General Population Regarding the Severity of Inflammation During the Acute Disease

The comparison of the results of FEV1, FVC, TLC, and DLCO and the severity of inflammation stratified by laboratory tests, including IL-6, CRP, and d-dimer concentration, revealed no significant differences regarding PFT parameters (Table 2). No statistically significant differences in PFT results were also found regarding the antiviral and anti-inflammatory treatment or immunotherapy used during the acute phase of COVID-19. Additionally, the results of Group II PFT were compared with the PFT in the anti-N IgG-negative group. No significant differences regarding PFT parameters were observed.

### 3.5. Assessment of the Associations Between Abnormalities of Chest CT and Respiratory Function Tests with Laboratory Parameters

Potential correlations were analyzed, although no clinically relevant associations were found (Table 6).

## 4. Discussion

Despite growing knowledge accumulated over the past three years regarding the long-term consequences of COVID-19, the course of recovery remains unpredictable, and no precise recommendations for monitoring convalescent patients can yet be formulated [6,7,18,19,20]. Although the clinical presentation of COVID-19 has evolved compared to the period dominated by the wild-type, Alpha, or Delta variants of SARS-CoV-2, the development of follow-up guidelines remains essential to ensure standardized patient care in everyday practice [21,22].

In most post-COVID-19 studies, follow-up assessments were conducted at approximately 3, 6, and 12 months after infection [3,5]. In most cases, lung function test results in patients recovering from COVID-19 improve over time, with the greatest improvement typically observed during the initial months following infection [7,23,24]. In individuals who experienced a moderate clinical course of COVID-19, improvements in pulmonary function tests began to plateau after 12 months [4]. Pulmonary function test results and chest imaging abnormalities may not correlate with the clinical symptoms reported by patients after COVID-19 [7,13,24].

First follow-up studies showed that reduced diffusion capacity is the most common abnormality in pulmonary function testing and depends on initial disease severity [3,11]. The more severe the course of the disease was, the lower the DLCO observed at follow-up [11]. Han et al. [6] and van Willingen [4] align with these observations. Impairment in pulmonary function is primarily associated with a reduced diffusing capacity for carbon monoxide (DLCO), which gradually improves at one, six, and twelve months following infection [4]. This process may be slower in older individuals, in those with more than three comorbidities (*p* < 0.001), and in patients who experienced a severe or critical course of the acute phase of the disease (*p* < 0.001) [4]. Van Willingen et al. suggest that pulmonary assessment should be prioritized, particularly in individuals who have experienced moderate to severe SARS-CoV-2 infection, within the first 6 months following recovery [4].

In Lee et al.’s systematic review and meta-analysis [3], reduced FVC and/or TLC were less frequent than impaired diffusion capacity. The prevalence of restrictive pulmonary dysfunction assessed by impaired FVC was lower in the longer-than-six-month follow-up studies [3].

Our study did not reveal significant differences in FEV1, FVC, TLC, and DLCO between COVID-19 convalescents 6 months after hospitalization and the general population. This may be attributed to the mild to moderate severity of the disease. None of the study participants required mechanical ventilation, and more than half received glucocorticoids for an average of 10 days. Additionally, pulmonary function tests were conducted 6 months after SARS-CoV-2 infection, a period during which previous studies have shown the most significant recovery of impaired pulmonary function. The initial severity of the disease serves as a guide for clinicians in assessing potential improvement and determining follow-up assessment strategies.

The ground-glass opacities and fibrotic pattern, including subpleural reticulations, traction bronchiectasis and honeycombing, and general architectural distortion, are mainly observed in chest CT scans after SARS-CoV-2 infection [5,6]. One of the first meta-analyses showed ground-glass opacity, linear opacities, and reticulation persist in over 30% of patients 6 months after COVID-19 [22]. Similar findings were observed after SARS infection. The pooled prevalence of persistent GGO and fibrotic pattern was 34% (95% CI 24–44%) and 32% (95% CI 23–40%) on follow-up chest CT performed 6 and 12 months after COVID-19. The prevalence did not decrease over time [3].

This finding corroborates other studies reporting that CT abnormalities following COVID-19 had resolved in 61% of participants (128 of 209) at three months and in 75% of participants (156 of 209) at 12 months [5]. However, Lalwani et al. showed that CT abnormalities are present after 12 months in 55.7% of participants [24]. In our study, chest CT conducted 6 months after COVID-19 revealed singular ground glass opacities in 20% of patients, thickening of interlobular septa and consolidations in 3.5% of patients, traction bronchiectasis and fibrosis in 5% of patients, and singular small nodular densities in 58% of patients.

The severe course of COVID-19 increases the risk of persistent changes in the lung parenchyma [3,22]. Interstitial lung disease with fibrosis is the most unfavorable outcome of COVID-19 [7]. Pan et al. pointed out that in addition to the proven relationship between ventilation and lung fibrosis, various other factors contribute to interstitial lesions, not only ARDS and mechanical injury. The researchers also noted the challenge of differentiating irreversible fibrosis from reversible interstitial abnormalities, which may lead to an overdiagnosis of fibrosis [5].

A prospective Swedish cohort study confirmed that the severity of COVID-19 was directly associated with greater impairment of lung function over the first year [11]. Previous studies identified similar risk factors for residual CT abnormalities at 1 year, including acute respiratory distress, age 50 years or older, and lymphopenia [5]. Non-severe and/or critical COVID-19 patients had less extent of recovery in comparison to the severe disease group. The researcher also observed a correlation between radiological abnormalities and reduced lung function [11].

Recent studies present 2-year observations after SARS-CoV-2 infection. Over one-third of COVID-19 convalescents have interstitial lung abnormalities present 24 months after infection. Han et al. [6] observed a fibrotic pattern of interstitial lung abnormalities in 23% of participants (33 of 144). Over 60% of participants showed complete radiologic resolution during the 2-year follow-up, with a statistically significant decrease observed at 6 months (*p* < 0.001). The researchers hypothesized that in patients with severe disease and critical respiratory failure, particularly those requiring ECMO treatment, a pattern of interstitial lung involvement characterized by simple restriction and reduced DLCO predominates [6]. Functional and subjective improvements are possible during the first year of follow-up.

Our study follow-up imaging revealed singular GGO in 20% of patients (52% of patients with GGO in baseline CT), occasional thickening of interlobular septa (3.5%) and consolidations (3.5%), and a traction bronchiectasis or fibrosis pattern in 5% of participants. Small nodular densities were observed in 58% of participants, but without clinical significance, requiring no additional diagnostic procedures. The almost complete resolution of CT findings could result from the mild to moderate course of the disease, as well as the average age of the participants, which was 52 years.

Based on the results of our study, we suggest that PFT should be considered the first step in post-COVID-19 follow-up assessment. The CT evaluation seems unnecessary in the case of normal PFT, and there are no other clinical indications or risk factors (e.g., tobacco smoking) for lung imaging tests. This strategy may help prevent unnecessary radiation exposure in COVID-19 survivors and conserve valuable CT resources.

During the COVID-19 pandemic, lung nodules were a common incidental finding in chest CT evaluation [25]. Management decisions regarding appropriate enrollment in lung cancer evaluation should be based on the nodule’s size, attenuation characteristics, and lung cancer risk factors [26,27]. No lung nodules were observed in the study group in chest CT, so no further cancer screening was necessary.

Many studies have been conducted to determine inflammatory markers that could predict the clinical course and prognosis of COVID-19 [28,29,30]. Not all markers that could potentially identify patient groups with an unfavorable disease course or poor prognosis for improvement are available in daily clinical practice [31]. The severe course of the SARS-CoV-2 infection is associated with coagulation and inflammatory markers, i.e., IL-6, D-dimer, CRP concentration, or neutrophil, lymphocyte, and PLT count [29,30,32]. Tan et al. [33] showed that CRP level was associated with CT scores and the development of COVID-19. Similar results were observed in an Italian study, which confirmed that CT involvement correlates with CRP, IL-6, IL-8, and tumor necrosis factor α (TNF-α) serum levels in severely ill patients with a high risk of acute respiratory failure [34]. Our study did not find any correlation between baseline chest CT results and respiratory function tests performed 6 months after COVID-19 in relation to selected laboratory tests, including PLT count, IL-6, and D-dimer concentration. The study participants did not require mechanical ventilation or admission to the intensive care unit, which may explain the lack of observed correlations.

Our study has several limitations. First, baseline pulmonary function tests performed at the end of the acute phase were unavailable. These baseline PFT values would help determine who should undergo follow-up tests after COVID-19, particularly in cases complicated by impaired respiratory function. Additionally, advanced cardiopulmonary exercise tests could provide more detailed information on the long-term consequences of COVID-19 in the general population. Second, the status of pre-COVID-19 chest imaging was not available. Furthermore, not all patients underwent follow-up CT scans (*n* = 118, 51%). Data from a larger cohort may help refine guidelines for further imaging follow-up, as only a small proportion of the population is likely to experience persistent changes in lung parenchyma. Finally, we acknowledge a potential assessment bias, as lung parenchymal involvement in COVID-19 pneumonia may have differed between subsequent pandemic waves.

## 5. Conclusions

- Most chest CT abnormalities resolved six months after recovery regardless of the SARS-CoV-2 variants. Routine imaging assessment may result in medically unjustified follow-up chest CT scans.
- Pulmonary function tests in patients recovered from COVID-19 do not differ from the results of the general population.
- Lung parenchyma involvement during the acute phase of infection and results of pulmonary function tests performed six months after recovery do not depend on the concentration of inflammatory parameters.

## Figures and Tables

**Table 1:** The demographic characteristics, selected laboratory tests, and applied treatment during hospitalization in Group I patients.

**Age**		
	Mean	50
	Min.–Max.	23–78
**Sex**		
	Male	117
	Female	115
**IL-6 (pg/mL)**		
Normal range < 7.0		
Mean		75.52
Min.–Max.		1.5–624
**CRP (mg/L)**		
Normal range: 0.00–5.00		
Mean		74
Min.–Max.		0.3–446.08
**D-dimer (ng/mL)**		
Normal range < 500		
Mean		1354
Min.–Max.		1–63,564
**WBC (10′3/µL)**		
Normal range: 4.00–10.00		
Mean		6.9
Min.–Max.		1.74–95.33
**PLT (10′3/µL)**		
Normal range: 150–300		
Mean		205
Min.–Max.		31–794
**Therapy (number of patients)**		
Remdesivir		41
Tocilizumab		29
Lopinavir + ritonavir		6
FFP		48
**Dexamethasone**		
Number of treated patients		126
Median dose (mg daily)		6
Median duration (days)		9
**Antibiotic (number of treated patients)**		
Levofloxacin		122
Ceftriaxone		39
Sulfamethoxazole + trimethoprim		2
Macrolides		9
Amoxicillin + clavulanic acid		2
Chloroquine		13
**Vaccinated (number of patients)**		113

CRP, C-reactive protein; IL-6, interleukin 6; PLT, platelet count; WBC, white blood cell count.

**Table 2:** Pulmonary Function Test Results.

Variable	Mean	Median	Min.	Max.	SD	Mean	Median	Min.	Max.	SD	*p*
	Group I *n* = 232					Group II *n* = 543					
**FEV1 (L)**	3.37	3.32	1.24	6.28	0.94	4.20	4.11	1.40	7.25	1.119	0.638
**% p.v.**	102	102	56	134	13.83507	101	102	46	146	13.52042	0.169
**FVC (L)**	4.22	4.22	1.62	7.82	1.18	3.33	3.28	1.14	5.87	0.91	0.908
**% p.v.**	101	101	47	135	13.51826	100	101	45	156	14.61207	0.996
**FEV1/FVC**	80.04	79.80	63.70	96.70	5.432	79.23	79.70	42.70	99.50	6.82036	0.159
**% p.v.**	100	100	79	124	6.92702	99	99	57	128	7.94838	0.004
**TLC (L)**	5.99	5.86	3.53	9.54	1.30	6.05	5.97	2.88	11.23	1.32648	0.569
**% p.v.**	99	98	65	149	12.45986	101	100	0.00000	160	14.70669	0.103
**DLCO (L)**	4.21	4.21	2.30	6.15	0.64	4.18	4.14	0.97	13.55	0.79251	0.395
**% p.v.**	72	72	39	102	10.82873	71	70	0.00000	229	13.93773	0.181
	**Group I a (IL-6 > 100 pg/mL) *n* = 36**					**Group I b (IL-6 < 100 pg/mL) *n* = 111**					
**FEV1 (L)**	3.37	3.44	1.54	5.19	0.94468	3.38	3.29	1.24	6.28	0.93133	0.977
**% p.v.**	102	102	59	126	12.83129	101	103	56	134	15.39898	0.824
**FVC (L)**	4.19	4.26	1.62	6.83	1.27	4.24	4.26	1.70	7.82	1.15437	0.950
**% p.v.**	99	102	47	131	14.42535	101	99	60	129	14.47683	0.747
**FEV1/FVC**	79.82	79.80	63.7	96.70	5.356	81.39	81.15	68.30	95.40	5.29543	0.102
**% p.v.**	100	100	79	122	6.64381	103	104	86	124	7.10940	0.019
**TLC (L)**	6.05	5.92	3.65	9.54	1.28	6.05	5.92	3.65	9.54	1.28066	0.744
**% p.v.**	98	98	65	143	12.51138	95	96	65	149	14.49745	0.158
**DLCO (L)**	4.18	4.18	2.3	5.74	0.64	3.92	3.96	2.73	4.75	0.58797	0.074
**% p.v.**	72	72	39	96	11.15244	70	70	49	92	11.04728	0.552
	**Group I c (PLT > 150 10′3/µL) *n* = 129**					**Group I d (PLT < 150 10′3/µL) *n* = 47**					
**FEV1 (L)**	3.36	3.29	1.54	6.28	0.92470	3.29	3.47	1.24	5.08	0.88502	0.842
**% p.v.**	102	102	59	134	12.93905	98	99	56	124	16.76967	0.222
**FVC (L)**	4.21	4.26	1.62	7.82	1.18587	4.12	4.22	1.70	6.54	1.11369	0.755
**% p.v.**	101	101	47	131	13.24848	97	98	60	125	15.43371	0.153
**FEV1/FVC**	80.19	80.40	63.70	96.70	5.70905	79.86	79.50	68.40	89.70	4.76430	0.719
**% p.v.**	101	101	79	124	7.18985	100	100	86	115	6.52758	0.753
**TLC (L)**	5.96	5.78	3.53	9.54	1.33434	5.95	5.98	3.65	8.03	1.16695	0.898
**% p.v.**	98	98	65	143	11.93104	97	95	65	149	13.67889	0.353
**DLCO (L)**	4.17	4.19	2.89	5.91	0.59064	4.12	4.20	2.30	5.74	0.73724	0.854
**% p.v.**	72	71	49	99	10.56927	71	72	39	95	11.48615	0.974
	**Group I e (d-dimer < 1000 ng/mL) *n* = 108**					**Group I f (d-dimer > 1000 ng/mL) *n* = 61**					
**FEV1 (L)**	3.33	3.28	1.24	5.19	0.83927	3.34	3.38	1.54	6.28	1.03635	0.861
**% p.v.**	102	102	56	134	14.18625	101	102	59	130	14.84052	0.967
**FVC (L)**	4.18	4.29	1.70	6.83	1.07	4.15	4.10	1.62	7.82	1.33964	0.596
**% p.v.**	101	100	60	131	13.61881	99	100	47	126	15.13241	0.672
**FEV1/FVC**	79.82	79.85	63.70	96.70	5.44787	80.91	80.90	69.70	95.40	5.09892	0.279
**% p.v.**	100	100	79	122	6.87570	102	102	86	124	7.01389	0.302
**TLC (L)**	5.99	5.92	4.02	8.63	1.19067	5.88	5.63	3.53	9.54	1.48830	0.472
**% p.v.**	99	98	75	149	11.66950	96	96	65	133	13.74404	0.295
**DLCO (L)**	4.24	4.26	2.89	5.91	0.61545	4.03	4.04	2.30	5.74	0.62203	0.065
**% p.v.**	73	73	48	99	10.81397	70	71	39	95	10.08383	0.027

DLCO, diffusing capacity of the lung for carbon monoxide (corrected for hemoglobin); FEV1, forced expiratory volume in the first second; FVC, forced vital capacity; p.v., predicted value; SD, standard deviation; TLC, total lung capacity.

**Table 3:** The medical and COVID-19 vaccination history of the study population.

	Group I *n* (%)	Group II *n* (%)
**Hypertension**	99 (42.7%)	157 (28.9%)
**Heart failure**	3 (1.3%)	12 (2.2%)
**Diabetes**	19 (8.2%)	39 (7.2%)
**Obesity**	102 (44%)	133 (24.5%)
**Renal insufficiency**	17 (7.3%)	30 (5.5%)
**Cancer**	24 (10.3%)	31 (5.7%)
**Chronic obstructive pulmonary disease**	8 (3.4%)	14 (2.6%)
**Asthma**	17 (7.3%)	26 (4.8%)
**Vaccination**	113 (49%)	188 (35%)

**Table 4:** The results of chest CT in two patients with persistent abnormalities in follow-up assessment.

Chest CT	Patient No. 1		Patient No. 2	
	Baseline	Follow-Up	Baseline	Follow-Up
**Ground glass opacity**	+	+	-	+
**Crazy** **paving pattern**	+	-	+	-
**Consolidation**	-	-	+	-
**Reticular pattern**	-	+	-	+
**Thickening** **of interlobular septa**	+	-	+	-
**Traction bronchiectasis**	-	-	-	-
**Air bronchogram**	+	+	-	-
**Bilateral distribution**	+	+	+	+
**Peripheral distribution**	+	+	+	-
**Diffuse lesion**	+	+	+	+
**Lung parenchyma involvement**	40%	1%	19/25	17/25

**Table 5:** The baseline CT findings depend on the laboratory test results and statistical significance during the acute phase of COVID-19 in Group I patients.

CT Findings	IL-6 (pg/mL)			PLT (10’3/µL)			d-Dimer (ng/mL)		
	>100	<100	*p*	PLT > 150	PLT < 150	*p*	>1000	<1000	*p*
**Ground glass opacity**	33/37 (89%)	7/10 (70%)	0.406	36/47 (77%)	10/12 (83%)	0.844	37/40 (92%)	10/16 (62%)	0.058
**Crazy paving pattern**	21/36 (58%)	9/10 (90%)	0.068	31/46 (67%)	8/12 (67%)	0.971	25/39 (64%)	12/16 (75%)	0.445
**Consolidation**	20/36	7/10		25/46	8/12		20/39	11/16	
			0.425			0.453			0.244
	(55%)	(70%)		(54%)	(67%)		(51%)	(69%)	
**Reticular pattern**	9/36 (25%)	1/10 (10%)	0.323	10/45 (22%)	4/12 (33%)	0.438	9/39 (25%)	5/16 (31%)	0.574
**Thickening of interlobular septa**	11/36 (30%)	3/10 (30%)	0.986	14/46 (30%)	2/12 (17%)	0.352	13/39 (33%)	3/16 (19%)	0.289
**Traction bronchiectasis**	0/36 (0%)	0/10 (0%)	-	0/46 (0%)	0/12 (0%)	-	0/39 (0%)	0/16 (0%)	-
**Air bronchogram**	2/36 (5%)	2/10 (20%)	0.164	5/46 (11%)	0/12 (0%)	0.238	1/39 (3%)	3/16 (19%)	0.042
**Bilateral distribution**	23/35	2/10 (20%)	0.754	28/45	9/12	0.420	23/39	12/16	0.271
	(64%)			(62%)	(75%)		(59%)	(75%)	
**Peripheral distribution**	3/36 (8%)	2/10 (20%)	0.311	4/45 (9%)	2/12 (17%)	0.450	4/39 (10%)	1/16 (6%)	0.637

**CT**, computed tomography; **IL-6**, interleukin 6; **PLT**, platelet count.

**Table 6:** Correlation between respiratory function tests and laboratory parameters.

	FEV1 (L)	FVC (L)	TLC (L)	DLCO (L)	IL-6 (pg/mL)	PLT (10’3/µL)	d-Dimer (ng/mL)
**FEV1 (L)**	1.00	0.96	0.69	0.23	0.06	−0.06	−0.08
**FVC (L)**	0.96	1.00	0.78	0.13	0.04	−0.06	−0.11
**TLC (L)**	0.69	0.78	1.00	−0.04	0.03	−0.06	−0.11
**DLCO (L)**	0.23	0.13	−0.04	1.00	−0.07	−0.03	−0.15
**IL-6** **(pg/mL)**	0.06	0.04	0.03	−0.07	1.00	−0.03	0.31
**PLT** **(10′3/µL)**	−0.06	−0.06	−0.06	−0.03	−0.03	1.00	0.06
**d-dimer** **(ng/mL)**	−0.08	−0.11	−0.11	−0.15	0.31	0.06	1.00

DLCO, diffusing capacity of the lung for carbon monoxide (corrected for hemoglobin). IL-6, interleukin 6; FEV1, forced expiratory volume in the first second; FVC, forced vital capacity; TLC, total lung capacity; PLT, platelet count.

## Data Availability

The dataset we generated and/or analyzed during the current study is not publicly available due to confidentiality issues but is available from the corresponding author on request.
